# Experimental study on the location of an acoustic emission source considering refraction in different media

**DOI:** 10.1038/s41598-017-07371-w

**Published:** 2017-08-07

**Authors:** Zi-long Zhou, Jing Zhou, Long-jun Dong, Xin Cai, Yi-chao Rui, Chang-tao Ke

**Affiliations:** School of Resources and Safety Engineering, Central South University, Changsha, 410083 China

## Abstract

The existing acoustic emission (AE) source location methods assume that acoustic waves propagate along straight lines, and the source location is determined by average wave velocity. Because of the heterogeneity of materials, location results often fail to meet the accuracy requirement. For this reason, an AE source location method considering refraction in different media was proposed in this paper. According to sensor coordinates, the arrival time of acoustic waves, the velocities of acoustic waves in two kinds of media, the space-time relation equations of the AE source point and the measuring point were established by the precise coordinates of the AE source based on Snell’s law. The feasibility of the algorithm was verified by experiments, and the factors influencing location accuracy were also analysed. The results show that the algorithm proposed in this paper is applicable for both the same medium and different media, and the accuracy of localization is not affected by the ratio of wave velocities in two media or the distance from the AE source to the refraction surface.

## Introduction

As a nondestructive testing method, the AE monitoring technique has found wide application in the rock mechanics field by providing solutions for parametric analysis, waveform analysis and AE source location^1–5^. This technique has proven to be very effective in detecting damage levels and determining the structural damage locations in rock. Accurate AE source location results can be used to infer crack initiation and propagation effectively. Therefore, improving the accuracy of the AE source location has always been a research focus^6–8^.

A substantial effort has been devoted to the study of acoustic emission source location, and a large number of algorithms were proposed. These algorithms can be divided into non-iterative algorithms and iterative algorithms. The non-iterative algorithms have to assume the same velocity for all stations, so they are inflexible in dealing with variable velocity models. Because of their flexibility in handling arrival time functions, iterative approaches are much more wildly used. The iterative algorithms include the derivative method, the sequential search method, the genetic algorithm and the simplex method^9, 10^. Ciampa and Meo applied wavelet analysis to determine wave velocity and then studied AE source location using the improved Newton iteration method^11^. Schumacher, Straub and Higgins proposed the Bayesian approach to improve the traditional Geiger’s method, and the new method could make uncertain parameters affect the location accuracy less^12^. Kao and Shan introduced the Source-Scanning Algorithm (SSA), which exploited waveform information without the need to calculate high frequency synthetic seismograms. It also required neither pre-assembled phase-picking data nor any a priori assumptions about the source geometry^13^. The sequential search method is restricted by the efficiency of searching, but the genetic algorithm and simplex method can solve this defect effectively. Kennett and Sambridge used the genetic algorithm for solving the problem of the local optimal solution of the traditional iterative method, ensuring that the global optimal solution could be found^14^. In 2005, Kim *et al.* used the genetic algorithm and two-point ray tracing in one-dimensional source location research. The results showed that this method nearly determined the exact source location without depending on initial velocity models^15^. In 2014, Kim, Hong and Kang introduced an iterative velocity updating scheme that could readily be combined with conventional hypocentral inversion methods^16^. Wang and Ge appled simplex method for AE source location, which showed good convergence in evaluating the high horizontal stresses for a limestone mine^17^. Li *et al.* also proposed an AE source location method based on the simplex method that directly searched for the AE source in the error space through four deformations of the simplex figures, and it was able to make use of both P-wave and S-wave velocities. The research showed that the simplex AE source location method could improve the accuracy and stability of the source location greatly when P-wave and S-wave velocities were simultaneously and correctly involved^18^.

All of the above mentioned methods, adopting whether the arrival time or arrival time difference as dependent variables on the basis of the assumption that acoustic waves spread along a straight line, are effective in locating the AE source in a single medium. However, these methods are not applicable to two or multiple media for refraction. Thus, there is a need to investigate an accurate location method that is applicable in two or multiple media. In the ground penetrating radar imaging field, Zhang, Wang and Wu proposed the constant method to determine the refraction point in multiple media based on an empirical formula^19^. In 2014, Zhang *et al.* applied the geometric mean method to locate the AE source in two media. This method has higher accuracy than the linear location method, but it used the empirical formula to solve the refraction point coordinates, which increases the positioning errors^20^. In 2015, Zhang, Wang and Jia further proposed the combined forward and inversion method, which has higher accuracy than the geometric mean method, but it is not suitable for the condition where AE sensors are far away from the hypocentre^21^. Kundu *et al.* proposed a new algorithm for accurately predicting the AE source location, such as an impact point on an anisotropic plate, but as this method of calculating the wave velocity is based on the derivation of a two-dimensional coordinate system, it may be hard to use it for the source location of three-dimensional anisotropic materials^22^. Gollob *et al.* found a novel multi-segment path analysis based on a heterogeneous velocity model for the AE source location in complex propagation media. This method considers the cracks and large air voids in concrete, which causes wave path deviations. However, it does not consider the change of path caused by refraction in a multi-layered medium^23^.

In this study, to improve the location accuracy in two media, an arrival time difference formula for AE source location is established considering the refraction of acoustic waves on the interface between two different media. A new AE source localization method considering the refraction in two media is proposed. Then, the test results from this method are compared with those from the traditional mixed wave velocity method. Finally, a parametric analysis is conducted to study the influence of different factors on location accuracy.

## Methods

### The principle of AE source localization based on the traditional method without considering the refraction

The traditional methods of AE source location are usually based on the time difference of arrival (TDOA), wave velocity and distances between sensors. They can be divided into the linear location, planar location and 3D location.

#### The principle of one-dimensional (1D) linear location

**Figure 1:**
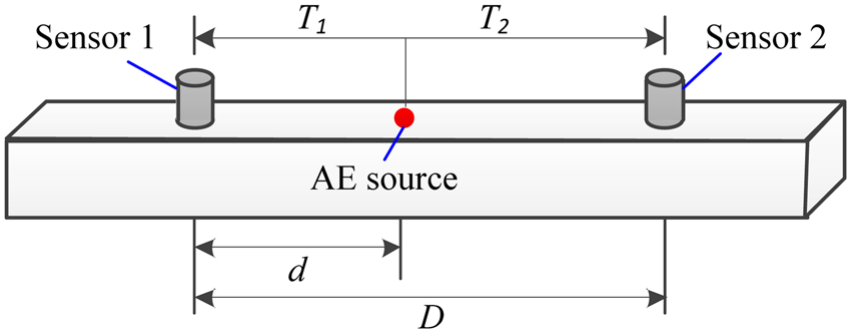
The schematic of the 1D linear location.

The linear location technique is suitable for rod-like media whose lengths are much larger than their widths, as shown in Fig. 1. Let the times when an AE signal arrives at AE sensors No. 1 and No. 2 be denoted by *T*_*1*_ and *T*_*2*_. Let the wave velocity be denoted by *V*. The times when an AE signal arrives at AE sensors from the AE source, also being called arrival time, can be detected by the AE sensor, and the difference of arrival times (Δ*T*) can be defined as

1 $${\rm{\Delta }}{T}_{2-1}={T}_{2}-{T}_{1}$$

where *T*_*1*_ and *T*_*2*_ are the arrival times detected by AE sensors No. 1 and No. 2, respectively.

Then, the distance between the AE source and AE sensor No. 1 (*L*_s−1_), which is closer to the AE source can be calculated as

2 $${L}_{s-1}=\frac{1}{2}(D-{\rm{\Delta }}TV)$$

where D is the distance between sensor 1 and sensor 2 and *V* is the wave velocity.

Then, the location of the AE source can be determined after the location of the AE sensors was measured directly.

#### The principle of two-dimensional (2D) planar location

**Figure 2:**
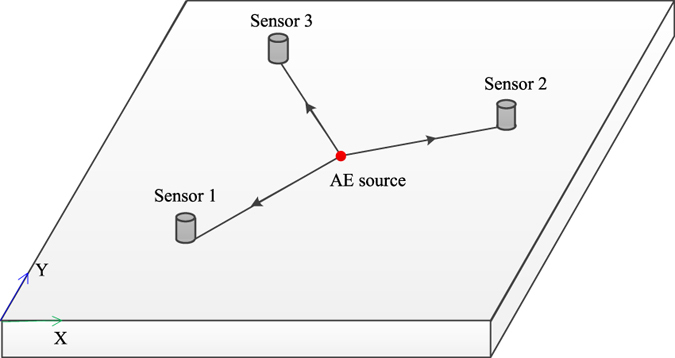
The schematic of the 2D planar location.

The principle of 2D planar location of an AE source is shown in Fig. 2. It is assumed that the AE source *S* (*x*_*s*_, *y*_*s*_) is located in a uniform medium. The AE sensors are located around the AE source, and the coordinates of an arbitrary sensor *I* is (*x*_*i*_, *y*_*i*_). The AE signal is produced at the initial moment of *T*_*0*_, and spread to any -sensor *I* at the moment *T*_*i*_.

The distance between the AE source *S* and sensor *I* can be obtained as follows:

3 $${L}_{s-i}=\sqrt{{({x}_{s}-{x}_{i})}^{2}+{({y}_{s}-{y}_{i})}^{2}}$$

For any sensor, the relationship between *T*_*0*_ and *T*_*i*_ can be described by:

4 $${T}_{i}-{T}_{0}=\frac{{L}_{s-i}}{V}$$

Because the initial time of the AE signal (T_0_) is difficult to measure accurately, we hope to eliminate T_0_ with the AE source localization as:

5 $${\rm{\Delta }}{T}_{i-j}={T}_{i}-{T}_{j}=\frac{{L}_{s-i}}{V}-\frac{{L}_{s-j}}{V}$$

According to the above relationships, it is possible to obtain two analytic solutions when there are three acoustic emission sensors that are not located on the same line. The correct coordinates of an AE source can be selected from these two analytic solutions in accordance with the actual situation, or further determined by increasing the number of AE sensors. Currently, the iterative algorithm is widely used in acoustic emission monitoring systems to calculate the numerical solution of a 2D planar location.

#### The principle of 3D location

**Figure 3:**
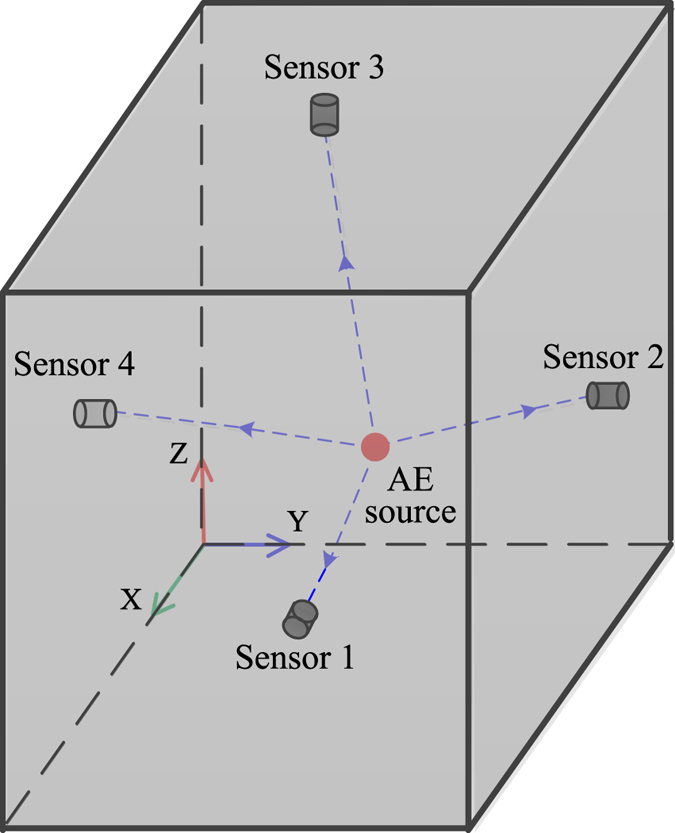
The schematic of the 3D TDOA location of an AE source.

In the principle of 3D location of an AE source, it is assumed that the AE source *S* (*x*_*i*_, *y*_*i*_, *z*_*s*_) is located in a uniform medium, as illustrated in Fig. 3. The AE sensors are located around the AE source, and the coordinates of an arbitrary sensor *i* are (*x*_*i*_, *y*_*i*_, *z*_*i*_). The distance between the AE source *S* and sensor *I* can be written as

6 $${L}_{s-i}=\sqrt{{({x}_{s}-{x}_{i})}^{2}+{({y}_{s}-{y}_{i})}^{2}+{({z}_{s}-{z}_{i})}^{2}}$$

For any sensor *I*, it has the following relationship

7 $${T}_{i}-{T}_{0}=\frac{{L}_{s-i}}{V}$$

The TDOA of any two sensors *I* and *J* can be described as follows:

8 $${\rm{\Delta }}{T}_{i-j}={T}_{i}-{T}_{j}=\frac{{L}_{s-i}}{V}-\frac{{L}_{s-j}}{V}$$

where *L*_*s*−*j*_ is the distance between the AE source *S* and sensor *J*.

According to the above equations, it is possible to obtain two analytic solutions when there are four acoustic emission sensors that are not located on the same plane. The correct solution can be selected from these two analytic solutions in accordance with the actual situation or increasing the number of AE sensors. The iterative algorithm is widely used in acoustic emission monitoring systems to calculate the numerical solution of a 3D location.

### The principle of AE source localization considering the refraction in different media

**Figure 4:**
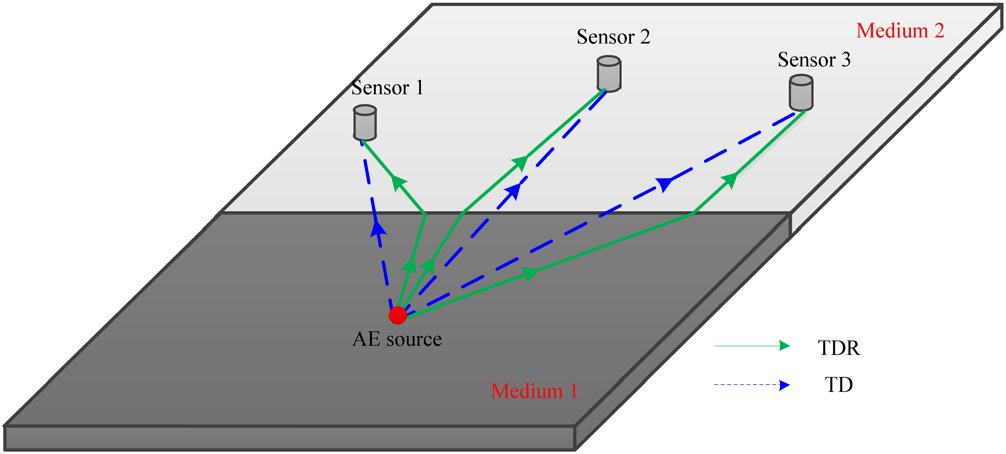
The propagation paths of the traditional location method and the new location method considering refraction.

When the AE source and sensors are located in two different media, the AE signal will be refracted at the interface between media. In this case, the results calculated by the traditional method of AE source localization without refraction are inaccurate. The propagation path of the traditional location method and the method considering refraction are shown in Fig. 4. The traditional method (TD) assumes that the acoustic wave still travels along a straight line in two different media, and the acoustic speed is the mixed velocity of the two different media. If the velocity difference between these two media is large, the location results will be inaccurate. Therefore, a new localization method considering refraction (TDR) is proposed in this study, and its principle of 3D location will be introduced in the following parts.

**Figure 5:**
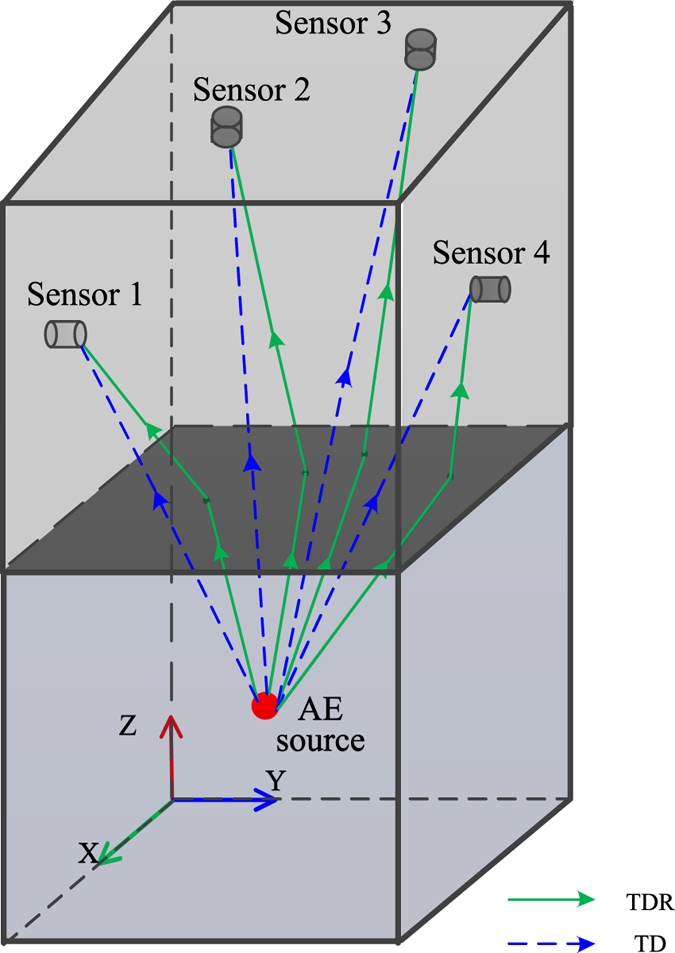
The propagation path of the 3D TDOA location method considering refraction.

As is shown in Fig. 5, the AE source and the AE sensors are located in medium *A* and medium *B*, respectively. It is assumed that the AE sensors are located at medium *B*, and the coordinates of them are (*x*_*i*_,*y*_*i*_, *z*_*i*_). The coordinates of the sound source *S* are (*x*_*s*_, *y*_*s*_, *z*_*s*_) The AE signal occurred at the AE source *S* at the initial moment *T*_*0*_. Then, the acoustic wave enters medium B from medium A while refraction occurs at the interface between the two media. Finally, the signal is received by the AE sensor at the moment *T*_*i*_. The refraction point *R* (*x*_*ri*_, *y*_*ri*_, *z*_*ri*_) is the intersection point of the path and the refracting surface. Then, the following relationships can be obtained.

The distance between the AE source *S* and the refraction point *R* can be determined by

9 $${D}_{s-ri}=\sqrt{{({x}_{s}-{x}_{ri})}^{2}+{({y}_{s}-{y}_{ri})}^{2}+{({z}_{s}-{z}_{ri})}^{2}}$$

The distance between the AE sensor *i* and the refraction point *R* can be obtained as follows:

10 $${D}_{i-ri}=\sqrt{{({x}_{i}-{x}_{ri})}^{2}+{({y}_{i}-{y}_{ri})}^{2}+{({z}_{i}-{z}_{ri})}^{2}}$$

For any sensor, there is the following relationship:

11 $${T}_{i}-{T}_{0}=\frac{{D}_{s-ri}}{{v}_{1}}+\frac{{D}_{i-ri}}{{v}_{2}}$$

where *v*_*1*_ and *v*_*2*_ are the wave velocities.

Since the initial time *T*_*0*_ is the same for all sensors, *T*_*0*_ can be eliminated by using any two sensors’ arrival times to subtract each other. The result is as follows:

12 $${\rm{\Delta }}{T}_{i-j}={T}_{i}-{T}_{j}=\frac{{D}_{s-ri}}{{v}_{1}}+\frac{{D}_{i-ri}}{{v}_{2}}-\frac{{D}_{s-rj}}{{v}_{1}}-\frac{{D}_{j-rj}}{{v}_{2}}$$

The difference between the experimental and the theoretical arrival time difference of the sensors *I* and *J* is defined as follows:

13 $$\hat{{\rm{\Delta }}}{T}_{n}=({t}_{i}-{t}_{j})-{\rm{\Delta }}{T}_{i-j}$$

where $({t}_{i}-{t}_{j})$ is the arrival time difference between times monitored by sensors *I* and *J*.

In practical applications, the only known parameters are *z*_ri_, *T*_i_, *T*_j_, (*X*_*i*_, *Y*_*i*_, Z_i_), *v*_1_ and *v*_2_. Therefore, the unknown quantities that need to be further determined are the source coordinates(*x*_s_, *y*_s_, *z*_s_), and the *x*_ri_ and *y*_ri_ of the refraction points’ coordinates.

At the same time, the refraction of an acoustic wave at the interface between two kinds of media should also meet Snell’s law:

14 $$\frac{\sin \,{\theta }_{1}}{\sin \,{\theta }_{2}}=\frac{{v}_{1}}{{v}_{2}}$$

15 $$\frac{{v}_{1}}{{v}_{2}}=\frac{|{x}_{s}-{x}_{i}|/\sqrt{{({x}_{s}-{x}_{i})}^{2}+{({y}_{s}-{y}_{i})}^{2}+{({z}_{s}-{z}_{i})}^{2}}}{|{x}_{i}-{x}_{ri}|/\sqrt{{({x}_{i}-{x}_{ri})}^{2}+{({y}_{i}-{y}_{ri})}^{2}+{({z}_{i}-{z}_{ri})}^{2}}},i=1,2,\cdots ,N$$

where ${\theta }_{1}$ and ${{\rm{\theta }}}_{2}$ are the incident and refracted angle, respectively.

Although the unknown parameters(*x*_s_,*y*_s_)and *x*_*r*i_ can be obtained by solving the simultaneous equations (12) and (15), it is too difficult to solve these complicated simultaneous equations. For convenience, the numerical method was employed to gain the coordinates of an AE source in this study. To verify the accuracy of the location algorithm proposed in this paper, the pencil-lead-break test was conducted, and the AE source locations were calculated by the new location method considering refraction. At the same time, the results of this new method were compared with that of traditional methods.

## Results

### The AE source location experiment in two different media

The experiment was carried out by an acoustic emission system. The AE source and the AE sensor were located in different media. The acoustic signal was generated by pencil-lead-break. According to the requirements of the pencil-lead-break experiment recorded in *Metal Pressure Vessel Acoustic Emission Testing and Result Evaluation (GB/T18182* —*2000)*, the size of HB pencil lead is 0.5 mm, and the pencil lead is broken at 30° to the surface of the rock specimen. If a signal’s spectral characteristic is within the range of 100~150 K, this signal will be considered as the effective signal.

#### The 2D planar location experiment considering the refraction in two media

**Figure 6:**
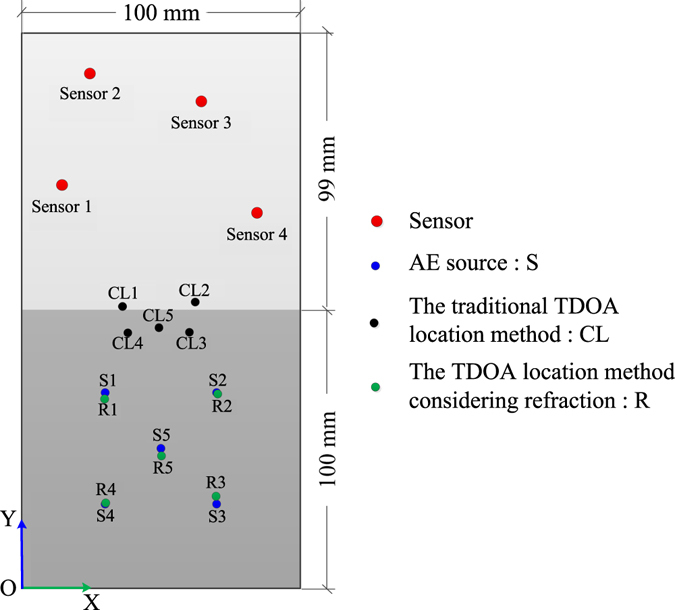
The results of the new and traditional methods in a 2D planar locating test.

The media used in the test were granite and iron specimens. The sizes of the granite and iron specimens were 99 × 100 × 20 mm, and the wave velocities were 4222.222 m/s for granite and 5657.143 m/s for iron, respectively. The mixing velocity of the two media was 4943.120 m/s based on the traditional method. In tests, four AE sensors were located on the iron specimen, and five AE sources occurred on the granite specimen. As shown in Fig. 6, the coordinates of the AE sensors are T_1_ (14.85, 154.45), T_2_ (34.65, 184.15), T_3_ (64.35, 174.25), and T_4_ (84.15, 144.55). In addition, the AE source coordinates are *S*_*1*_ (30, 70), *S*_*2*_ (70, 70), *S*_*3*_ (70, 30), *S*_*4*_ (30, 30), and *S*_*5*_ (50, 50).

**Figure 7:**
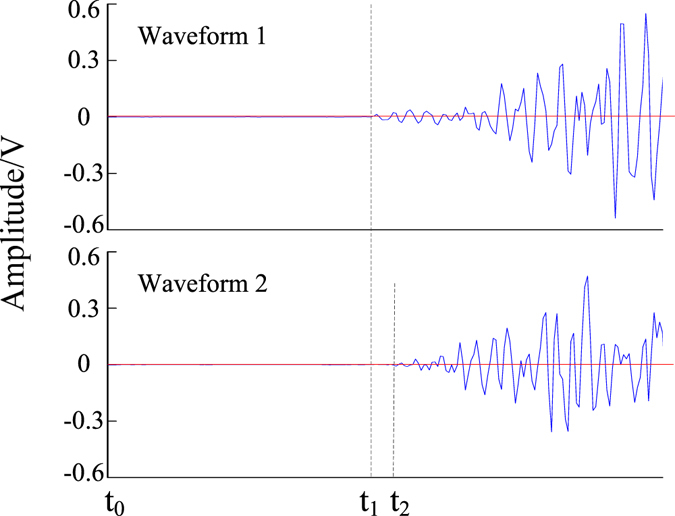
The diagram of detecting the time arrival data.

**Figure 8:**
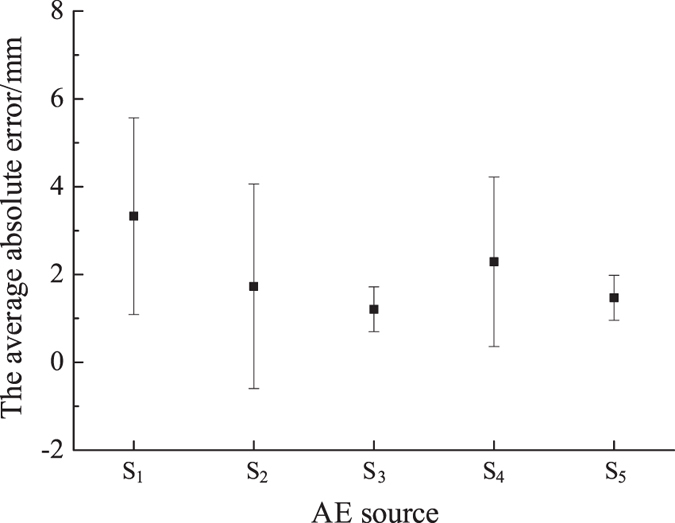
The average absolute errors of the AE source in the 2D location test.

According to the *Acoustic Emission Testing and Evaluation of Metal Pressure Vessels*, the pencil leads were broken at the AE source points that had been marked on the granite specimen in advance. The repeated experiments were done at each AE source point, then the three groups of data that meet the requirements of the standard were selected as the experimental data. One of the waveforms is shown in Fig. 7. The time arrival data were detected according to the waveform diagram. The signal of a pencil-lead-break arrived at the sensor 1 and sensor 2 at *t*_*1*_ and *t*_*2*_, respectively. The plane location was calculated according to the method proposed in this paper, and by the traditional location method for comparison. As shown in Fig. 6, the coordinates of the AE sources, which were solved by the new and traditional location methods, are plotted on the two-dimensional coordinate system. It can be seen that the location of the AE source can be calculated by the new location method proposed in this paper, and the accuracy of the new proposed method is better than the traditional method. The average absolute errors and their standard deviations are shown in Fig. 8. It can be seen that the average absolute errors are less than 4 mm. The algorithm proposed in this paper is steady according to the standard deviations.

#### The 3D location experiment considering the refraction in two media

**Figure 9:**
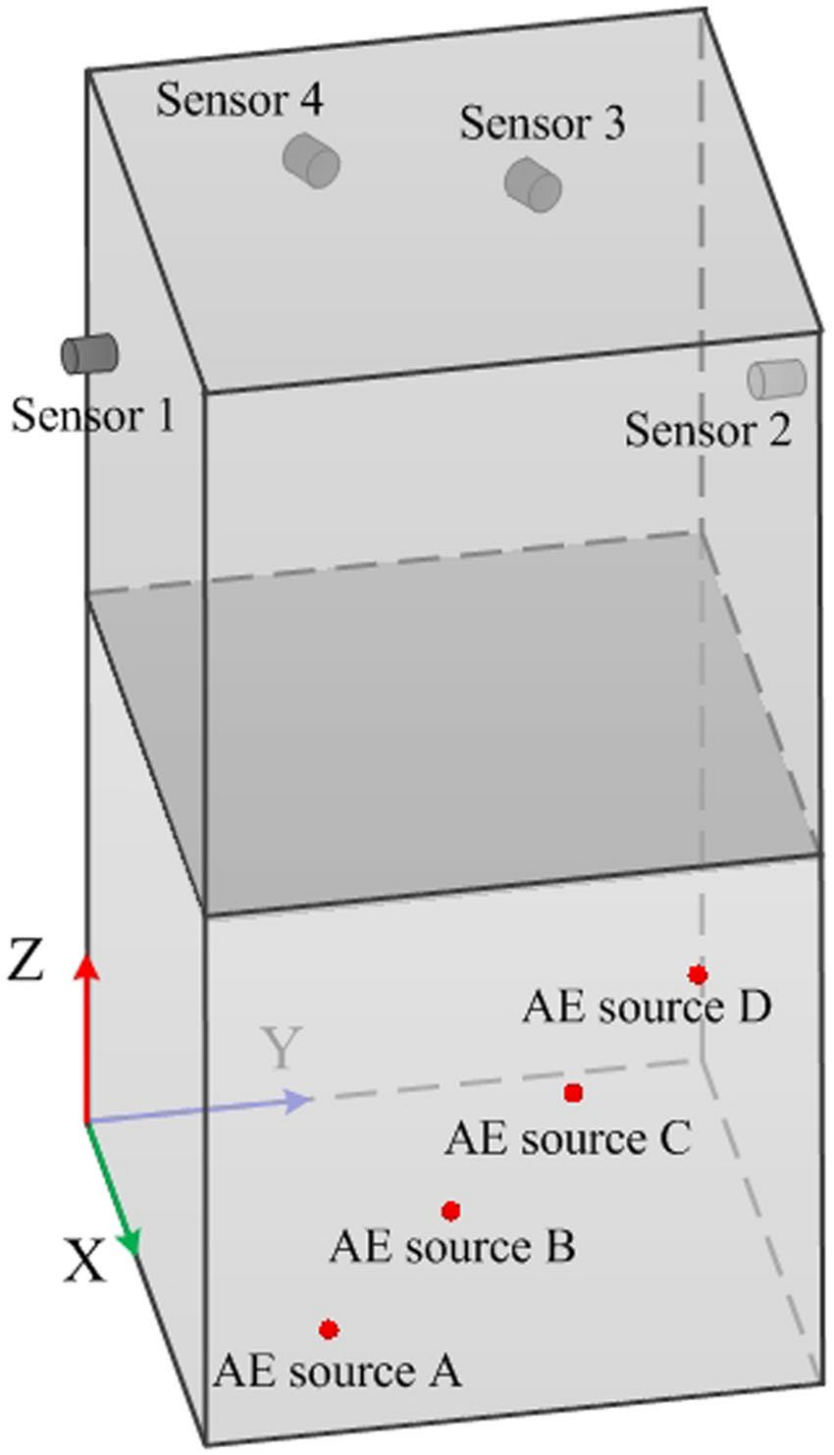
The positions of the sensors and the pencil-lead-break positions of the test.

The positions of the AE sensors and the AE sources are shown in Fig. 9. The sizes of the granite and iron specimens were 99 × 100 × 100 mm, and the wave velocities were 4222.222 m/s for granite and 5657.143 m/s for iron, respectively. The mixing velocity of the two media was 4943.120 m/s based on the traditional method. Four sensors should not be placed in the same plane. Thus, the coordinates of the sensors are NO. 1 (14.85, 0, 154.45), NO. 2 (44.55, 100, 154.45), NO. 3 (0, 74.25, 164.35) and NO. 4 (0, 34.65, 174.25). The AE source coordinates are A (99, 20, 20), B (99, 40, 40), C (99, 60, 60), and D (99, 80, 80), respectively.

**Figure 10:**
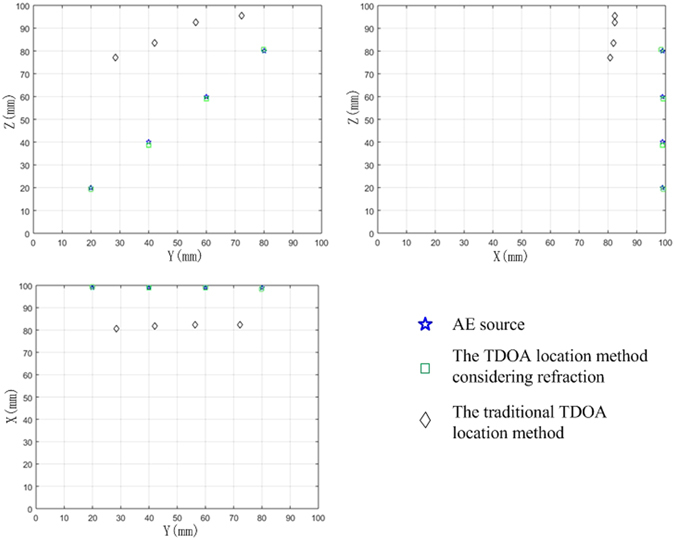
The results of the new and traditional methods in a 3D locating test.

**Figure 11:**
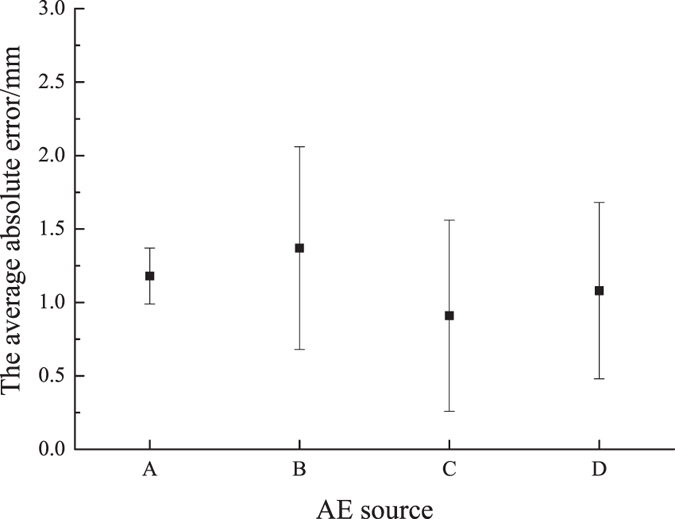
The average absolute errors of the AE sources in the 3D location test.

The operation method of the 3D location test is the same as the 2D planar test. The time arrival data were detected according to the waveform diagram. The position was carried out according to the TDR proposed in this paper. At the same time, the TD was used for comparison. The results of the two methods are shown in Table 1, Figs 10 and 11. The results are the average of three repeated experiments. The absolute error in Table 1 is the distance between the average location points and the corresponding pencil-lead-break point.

**Table 1:** The results of the new and traditional methods in a 3D locating test.

AE source	Method	X	Y	Z	Absolute error/mm
A	AE source coordinates	99	20	20	
	TDR	99.17	19.97	19.43	0.6
	TD	80.67	28.5	77.1	60.57
B	AE source coordinates	99	40	40	
	TDR	99.07	40.07	38.63	1.37
	TD	81.9	41.97	83.57	46.85
C	AE source coordinates	99	60	60	
	TDR	99.1	60.1	59.13	0.88
	TD	82.3	56.37	92.47	36.69
D	AE source coordinates	99	80	80	
	TDR	98.53	79.93	80.63	0.79
	TD	82.27	72.23	95.5	23.34

It can be seen that the absolute distance error is less than 2 mm and stable when refraction is considered. However, the lowest absolute distance error is as high as 23.34 mm, and the error is unstable when locating using the traditional method. The difference between the maximum absolute distance error and the minimum absolute distance error is 37.23 mm. Therefore, it is not feasible to use the traditional method in this situation.

Figure 11 gives the average absolute error and their standard deviations. It can be seen that the average absolute error is less than 1.5 mm, which proves this algorithm proposed in this paper can satisfy the precision requirement and it is stable.

## Discussion

A numerical model was developed to analyse the factors influencing the location accuracy. The numerical simulation model is composed of two cubes made of different materials. The cube on the top has the dimensions of 99 × 100 × 100 mm, and the corresponding wave velocity is 2000 m/s. The cube below has the dimensions of 99 × 100 × 99 mm, and the corresponding wave velocity is 4000 m/s. Five AE sources, one is located inside the lower cube and the other four are located on the outside surface of the lower cube. Their coordinates are E (49.5, 50, 50), F (0, 50, 20), G (49.5, 100, 40), H (99, 50, 60), and I (49.5, 0, 80).

It must be noted that all AE sensors are not located in the same plane. The coordinates of the four AE sensors are No. 1 (14.85, 0, 154.45), No. 2 (44.55, 100, 154.45), No. 3 (0, 74.25, 164.35), and No. 4 (0, 34.65, 174.25).

According to the given wave velocities in the two media and the location of the AE sensors, the locations of the five AE sources were determined by the method proposed in this paper. At the same time, the traditional linear method with mixed wave velocity was also adopted to locate the AE source for comparison. The results are shown in Table 2.

**Table 2:** Results of the location and absolute error of distance.

AE source	Methods	X	Y	Z	Absolute error/mm
E	AE source coordinates	49.50	50.00	50.00	
	TDR	49.50	50.00	50.40	0.40
	TD	44.00	48.80	101.00	51.31
F	AE source coordinates	0.00	50.00	20.00	
	TDR	0.00	49.80	21.60	1.61
	TD	45.00	48.50	105.50	96.63
G	AE source coordinates	49.50	100.00	40.00	
	TDR	49.40	99.90	40.50	0.52
	TD	43.80	73.00	106.30	71.81
H	AE source coordinates	99.00	50.00	60.00	
	TDR	98.00	49.90	60.20	1.02
	TD	70.00	48.10	113.40	60.80
I	AE source coordinates	49.50	0.00	80.00	
	TDR	48.40	0.00	79.50	1.21
	TD	44.60	21.80	112.90	39.77

**Figure 12:**
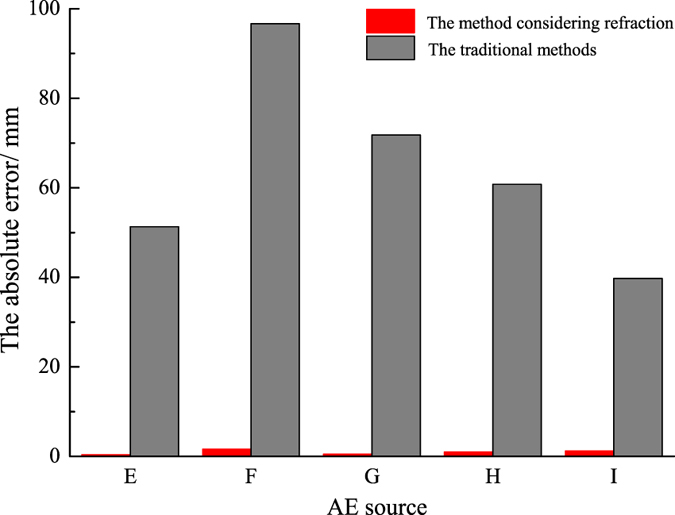
The absolute errors of the two algorithms.

As can be seen from Table 2, the AE sources, which are located inside the specimen and on the outside surface of the specimen, can all be precisely located with the method considering refraction. In Fig. 12, it is obvious that the absolute error is approximately 1 mm when calculated by the new method, compared to that by the mixed wave velocity method, whose absolute error can even reach from 39.77 mm–96.63 mm.

**Figure 13:**
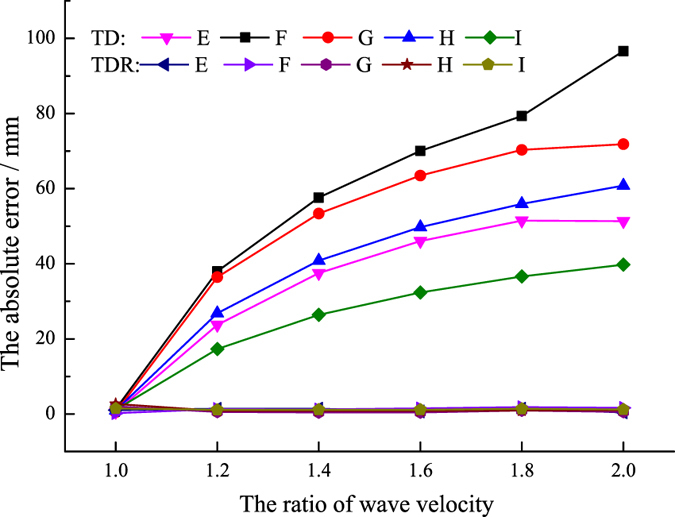
The dependency level of the location results on wave velocity accuracy.

In practical application, it is difficult to measured accurate wave velocity. Therefore, the influence of the measuring error of wave velocity on the precision of location results was analysed. The arrival time and other parameters are constant, and only the wave velocity is changed in the range between 1% and 5%. The dependency of the location accuracy on wave velocity is shown in Fig. 13. The results show that the proposed method and the traditional method have the same tendency of dependency. With the variation of wave velocity increasing, the location error gets larger.

**Figure 14:**
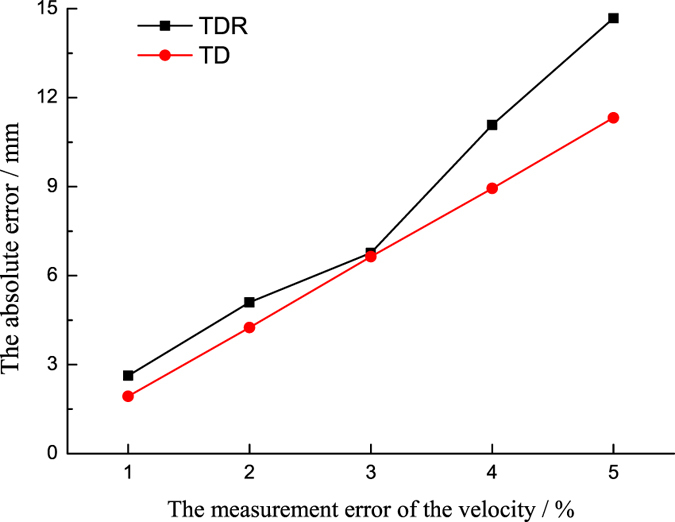
The location errors with different ratios of wave velocity.

Using the proposed innovative method, the influence of the wave velocities ratio in the two media on location accuracy was also analysed. When the ratio of the wave velocities changed from 1.0 to 2.0, the absolute errors from the new method and from the traditional method are shown in Fig. 14. It can be seen that the absolute error from the traditional mixed wave velocity method increases with the ratio of the wave velocities. The results indicated that the traditional mixed wave velocity method cannot precisely locate the AE sources when the ratio of the wave velocities in two mediums is large. However, the absolute error from the new method is always approximately 1 mm when the ratio of the wave velocities increases from 1.0 to 2.0, which means that the new method has a good feasibility in different cases.

**Figure 15:**
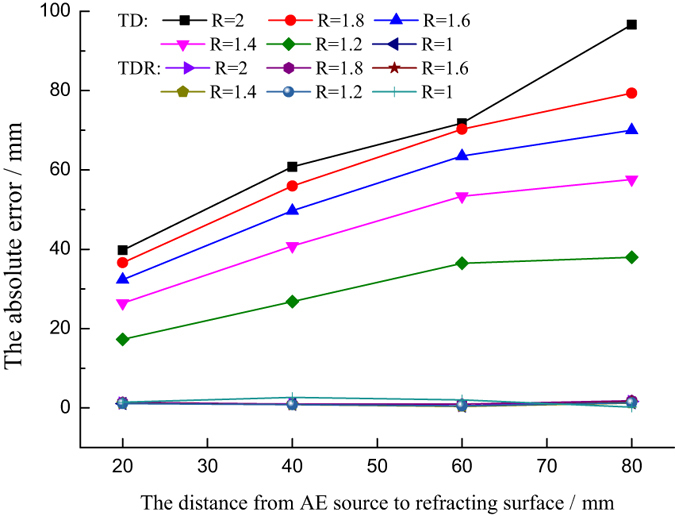
The influence of the distance from the sound source to the refracting surface on location error.

Figure 15 shows the influence of distance from the AE source to the refracting surface on location accuracy. It can be seen that the new method is not influenced by the distance from the AE source to the refracting surface. However, the traditional mixed wave velocity method is significantly influenced by this factor, especially when the ratio of the wave velocities in two media is different. The tendency of variation is the same when the ratio of wave velocities is different. The further from the refracting surface, the larger the absolute error.

It needs to mention that, the materials in daily life may be very complex with multiple layers, mixed material constitution, and complex shape or cross-sections. These are the vital factors that cause the traditional AE source location method less accuracy. In the traditional method, all these factors were ignored by using a mixed velocity.

**Figure 16:**
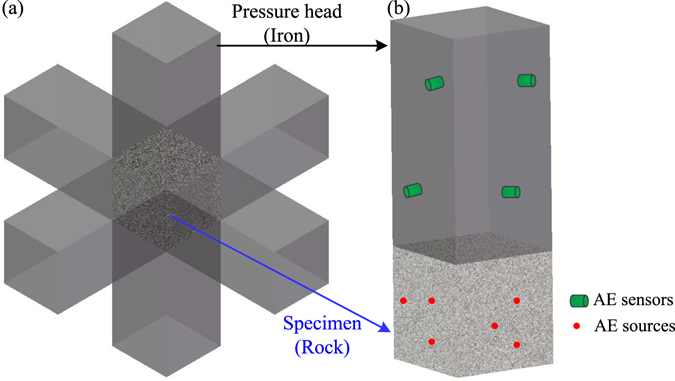
The triaxial compression test of rock. (**a**) The layout of the pressure heads and the specimen. (**b**) The AE source location from the pressure heads.

In this study, the non-uniform of the material was considered and idealized as the wave refraction at the interface of different medium. This proposed method can be used in real life, such as the AE source location for triaxial compression test^24^. In the triaxial test, the pressure heads and specimen are arranged as Fig. 16a. During the procedure of AE source location, the acoustic signals emit from the rock specimen, refract at the interface between the specimen and the pressure head, and reach the AE sensors. The new AE source location method proposed in this paper can be used for this case as Fig. 16b. This previous analyses showed that the new method considering the refraction between the specimen and the pressure head would be much more reasonable than the traditional method using a mixed velocity. Of course, this method is now applicable to the case that both media have symmetrical and linear cross-sections. For very complex media or structures in daily life, the further study is needed to verify the applicability of the proposed method.

## Conclusion

An AE source location method considering refraction at the interface between two media was proposed in this paper. This method is based on the assumption that acoustic waves spread along a straight line in a single isotropic medium and would refract at the interface between two media. The sum of squares of absolute error between the actual arrival time and the theoretical arrival time is taken as an objective function. Then, the optimal solution is solved by this function. Moreover, the influence of factors on the locating accuracy for the new and traditional methods is compared and discussed. The conclusions can be drawn as follows:

1. The proposed innovative location method is applicable for both the same medium and different media. In addition, the proposed method has a greater advantage over the traditional method in location accuracy and stability.
2. The location accuracy of the proposed and the traditional algorithm can be affected by the accuracy of wave velocity measurement. The accuracy for traditional method would decrease when the wave velocity difference increases, but that for the proposed method is more stable.
3. The proposed method is not affected by the distance from the AE source to the refracting surface. In contrast, the traditional method is affected by this distance significantly. The locating accuracy decreases with the increase in distance.
4. The new proposed AE source location method, considering refraction at the interface between two different media, is much more reasonable than the traditional one using mixed velocity assumption. Of course, for very complex media, further effort is needed to verify the applicability of the proposed method.
